# PD-1 high expression predicts lower local disease control in stage IV M0 nasopharyngeal carcinoma

**DOI:** 10.1186/s12885-019-5689-y

**Published:** 2019-05-28

**Authors:** Feng Jiang, Wei Yu, Fanrui Zeng, Guoping Cheng, Jing Xu, Shifeng Yang, Yongjie Shui, Dang Wu, Xiao-fang Yu, Qichun Wei

**Affiliations:** 1 Department of Radiation Oncology, The Second Affiliated Hospital, Zhejiang University School of Medicine, Jiefang Road 88, Hangzhou, 310009 People’s Republic of China; 2 Ministry of Education Key Laboratory of Cancer Prevention and Intervention, Zhejiang University School of Medicine, Hangzhou, 310009 People’s Republic of China; 3 Department of Radiation Oncology, Zhejiang Cancer Hospital, Hangzhou, 310022 People’s Republic of China; 4 Department of Pathology, Zhejiang Cancer Hospital, Hangzhou, 310022 People’s Republic of China

**Keywords:** Nasopharyngeal carcinoma, PD-1/PD-L1, Local recurrence, Prognosis

## Abstract

**Background:**

Tumor-infiltrating lymphocytes (TILs) play a critical role in tumor immune surveillance and immune suppression. Understanding tumor infiltrating T cell subset density, location and PD-1/PD-L1 expression might provide insight for the prediction of tumor therapeutic response and clinical outcome. The purpose of this study was to evaluate the expression and localization of CD8, FoxP3, PD-1, and PD-L1 in primary tumor tissues and their effects on prognosis of stage IV M0 locally advanced nasopharyngeal carcinoma (NPC) patients.

**Methods:**

Sixty NPC patients with stage IV M0 locally advanced disease were treated with definitive chemoradiation. Tumor biopsies from primary lesion were analyzed for the expression and localization of CD8, FoxP3, PD-1, and PD-L1 by immunohistochemistry. Their associations with local disease control and survival of NPC were analyzed.

**Results:**

The average follow-up time was 43 months (range from 14 to 61 months). High expression of CD8^+,^ FoxP3^+^, PD-1^+^ and PD-L1^+^ was observed in 60, 86.7, 56.7, and 91.7% of patients, respectively. There was no correlation between clinicopathological features and the expression of these immune markers. High PD-1 expression was found to be associated with lower local disease control (5-year LRFS 23.2% vs 96.8%, *p* < 0.001) and unfavorable clinical outcome (5-year OS 47.4% vs 73.3%, *p* = 0.014). In multivariate analysis, PD-1 expression was also an adverse prognostic factor for 5-year OS (HR: 3.68, *P* = 0.023) and LRFS (HR: 16.89, 1.27–11.84, *P* = 0.007). Those with PD-1 distribution in both stroma and tumor region had the poorest prognosis. However, PD-1 expression has no significant correlation with 5-year RRFS (*p* = 0.980) and DMFS (*p* = 0.865). Patients with both PD-1 and PD-L1 high expression had significant poor local disease control (5-year LRFS 96.0% vs 43.0%, *p* < 0.001) and overall survival (5-year OS 80.8% vs 45.1%, p < 0.001) compared with the others. Other immune markers were not found having corrections with disease control and survival.

**Conclusions:**

PD-1 high expression, especially with PD-L1 co-expression, is associated with high local recurrence and unfavorable clinical outcome for stage IV M0 NPC patients, and might be a potential target for immunotherapy.

## Background

Nasopharyngeal carcinoma (NPC) is quite different from the head-and-neck squamous carcinomas (HNSCC) by its epidemiology, pathology, and high sensitivity to treatment [1]. Intensity-modulated radiotherapy (IMRT) is a major breakthrough in the treatment of NPC which can produce highly conformal dose distributions with steep dose gradients over the conventional 2D radiotherapy [2, 3]. As a result, encouraging outcomes of NPC patients has been reported with an over 80% 3-year overall survival (OS) and over 90% 3-year local disease control (LDC**)** [4–6]. However, the result of patients with locally advanced disease, especially stage IV M0, is still disappointing. In our previous report [7], the 5-year progression-free survival (PFS) for those stage IV M0 patients was only 68.2%. Similar outcomes were also seen in other reports [8, 9]. More effectively novel treatment is urgent to be developed. Identifying patients with high risk of therapeutic failure will contribute to improve the efficacy of therapy.

Increasing evidence indicates that tumor-infiltrating lymphocytes (TILs) play a critical role in tumor immune surveillance and immune suppression [10]. It has been reported that the type, functional orientation, density, and location of immune cells in the context of continuum of cancer immunosurveillance are all associated with patient survival [11]. For instance, CD8^+^ cytotoxic T lymphocytes directly mediate tumor cell death through producing cytotoxic granules including perforin and granzymes, and have been linked with favorable prognosis and treatment response of multiple malignancies [12, 13]. On the contrary, FoxP3^+^ regulatory T lymphocytes (Tregs) are supposed to mediate immune tolerance and associated with therapeutic failure and poor cancer survival [14–16]. Recently, the immune checkpoint programmed death-1 (PD-1) is characterized as a hallmark of T cell exhaustion [17]. PD-1 mediates immune evasion through binding with its ligand, programmed death-ligand 1 (PD-L1), which expressed on tumor cells, stromal cells, and some myeloid cells. The expression of PD-1/PD-L1 has been reported to be prognostic factors in several malignancies [18, 19], including NPC [20–22]. However, PD-1 expresses on CD4^+^ T cells, γδ T cells and tumor associated macrophages (TAMs) in addition to CD8^+^ T cells [23–25], mediating extensive immunosuppression in tumor microenvironment (TEM). Therefore, further study evaluating the immunosuppressive state of NPC microenvironment, especially PD-1 expression and localization, is needed. Understanding tumor infiltrating T cell subset density, location and PD-1/PD-L1 expression will provide insight for the prediction of tumor therapeutic response and clinical outcome.

In this study, the expression and localization of CD8, FoxP3, PD-1, and PD-L1 in NPC tumor tissues was evaluated by immunohistochemical staining. Their association with disease control and survival has been analyzed.

We have demonstrated that PD-1 high expression, especially with PD-L1 co-high expression, correlates with higher local recurrence and poorer overall survival in NPC patients with stage IV M0 disease. Thus PD-1 may be a prognostic biomarker for local recurrence and unfavorable survival. Our study implies that anti-PD-L1/PD-1 axis would be a promising therapeutic approach for advanced NPC patients.

## Methods

### General information

This study was approved by the Institutional Human Experiment and Ethics Committee of Zhejiang Cancer Hospital. Written informed consent was obtained from all the patients regarding the data and samples to be used for research. Sixty NPC patients underwent radical treatment with 7th AJCC stage IVa-b disease was identified by reviewing the hospital database during January 2008 and December 2010. Clinical data and primary tumor samples were retrospectively studied. Among the 60 enrolled patients, 47 males and 13 females, the median age was 47 years old (range: 19–72). Fifty-seven patients were classified as non-keratinizing phenotype and three as keratinizing squamous carcinoma. All patients were treated with radical IMRT combined with chemotherapy and then followed up regularly. Treatment details were described in our previous work [7]. All patients were treated with definitive IMRT. The prescribed dose was 69 Gy to GTV, 60 Gy to CTV-1 (high risk regions of primary and positive node regions) and 54 Gy to CTV-2 (other node regions) in 30 fractions, one fraction daily over 5 days/week. Neoadjuvant or adjuvant chemotherapy consisted of cisplatin with 5-fluorouracil or cisplatin with docetaxel and 5-fluorouracil was given every 3 weeks for three cycles, concomitant cisplatin 80 mg/m2 was given on day 1 and 22 of radiotherapy.

### Immunohistochemistry

We used the methodology previously described by our esophageal team [26]. The nasopharyngeal tumor samples at diagnosis were fixed with formalin and embedded into paraffin using a tissue processor. Standard immunohistochemical analysis was performed with the primary antibody against human CD8 (Novocastra Leica Biosystems, clone 4B11), PD-1 (Abcam, clone NAT105), PD-L1 (Sigma-Aldrich, clone SAB2900365) and FoxP3 (Cell Signaling Technology, clone D2W8E) at a dilution in 1:200, 1:100, 1:400 and 1:100, respectively.

Immunohistochemistry results were evaluated by scanning each slide under low-power magnification (× 100) to identify regions containing positive immunoreactivity. Immunostainings were further evaluated at high-power magnification (× 400). TILs were shown in H&E-stained TMA slides and the cells were counted independently by two pathologists under the entire visual region. Then positive area was evaluated in each histospot. Mononuclear cells around tumor nests and not directly contacting carcinoma cells was categorized as stromal TILs, and those within tumor nests directly interacting with carcinoma cells as intratumoral TILs. About 400 cells in a high-power field were evaluated, and repeated in another field. Cells stained with any intensity level were defined as positive. PD-1, CD8, and FoxP3 expression was scored as the percentage of positive TILs. While PD-L1 expression was scored as the percentage of positive tumor cells (TCs). Doubtful cases were discussed by the two pathologists until consensus was achieved.

### Statistical analysis

Patients were divided as high and low expression group according to the percentage of positive expression cells of bio-markers. Optimal cut-off point for each marker was determined by using the X-Tile statistical package (Yale University, New Haven, CT) basing on the treatment outcomes [27].

Statistical analyses were performed using statistics software (version 18.0; SPSS, Chicago, IL). Chi-square test was used to assess the expression of biomarkers correlated with clinical parameters. Survival curves were generated by Kaplan-Meier method, and the significance of differences were determined by the log-rank test. Multiple variable analysis was performed by Cox proportional hazards model. *P*-value < 0.05 in a two-tailed test was considered statistical significance.

## Results

The average follow-up time was 43 months (range from 14 to 61 months). In this cohort, 30 patients encountered disease failure (local, regional, and distant relapse in 14, 4 and 15 patients, respectively). Eighteen patients died during the follow-up, 17 of them due to disease progression.

### Expression and localization of CD8, FoxP3, PD-1, and PD-L1 in tumoral and stromal regions of NPC tumors

**Fig. 1:**
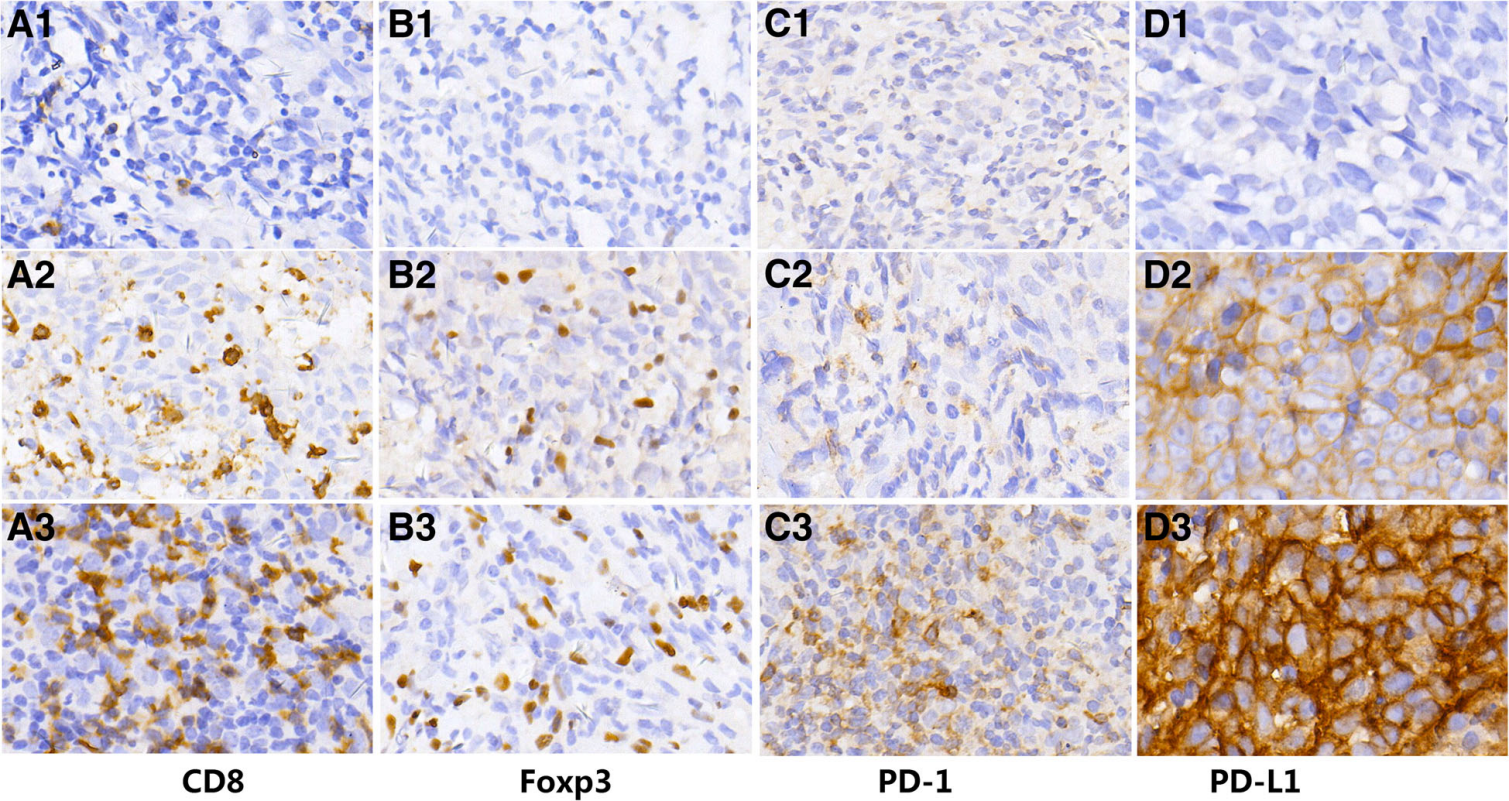
Representative images of expression pattern for CD8, FoxP3, PD-1 and PD-L1

Sixty tumor sections were stained with anti-CD8, anti-FoxP3, anti-PD-1, and anti-PD-L1 antibodies. The representative images were shown in Fig. 1. Then the intratumoral and stromal cell localization was then quantified. Results indicated that the large majority of CD8^+^, FoxP3^+^, and PD-1^+^ cells were located within the stroma, while PD-L1^+^ cells were mainly located within intratumoral region.

By the X-Tile package using disease-free survival as the end-point, the optimal cut-off point was 5% for PD-1 expression on TILs and 1% for PD-L1 expression on TCs. The corresponding cut-off values for CD8 in stroma and tumor region was 40 and 2%, while 1% for Foxp3 in both stroma and tumor region. Then the patients were grouped as high or low expression by optimal cut-off value. High expression of CD8, FoxP3, PD-1 and PD-L1 was observed in 60.0, 86.7, 56.7, and 91.7% of patients, respectively. The association of these markers with clinical characteristics in stage IVa-b NPC patients was shown in Table 1, including patient’s gender, age, pathology, and TNM staging. There was no correlation between all these parameters and the expression of CD8, FoxP3, PD-1, and PD-L1.

**Table 1:** Clinicopathologic variables and the expression of CD8, FoxP3, PD-1, and PD-L1 in stage IVa-b NPC patients

Variables		n	PD-1 high	p	PD-L1 high	p	CD8 high	p	FoxP3	p
Total		60	34 56.7%		55 91.7%		36 60.0%		52 86.7%	
Gender	male	47	26 55.3%	0.760	42 89.4%	0.575	28 59.6%	0.988	39 83.0%	0.150
	female	13	8 61.5%		13,100%		8 61.5%		13,100%	
Age	<medial	28	15 53.6%	0.795	25 89.3%	0.657	20 71.4%	0.208	26 92.9%	0.228
	≥medial	32	19 59.4%		30 93.8%		16 50.0%		26 81.3%	
Pathology	WHO I	3	2 66.7%	1.000	3100%	1.000	0 0%	0.337	3100%	0.566
	WHO II	57	32 56.1%		55 91.7%		33 57.9%		49 85.9%	
T stage	T1–2	4	1 25.0%	0.346	4100.0%	0.695	3 75.0%	0.314	3 75.0%	0.446
	T3	12	8 66.7%		11 91.7%		6 50.0%		12,100%	
	T4	44	25 56.8%		40 90.9%		27 61.4%		37 84.1%	
N stage	N1	18	11 61.1%	0.310	16 88.9%	0.853	13 72.2%	0.317	16 88.9%	0.489
	N2	25	16 64.0%		23 92.0%		15 60.0%		22 88.0%	
	N3	17	7 41.2%		16 94.1%		8 47.1%		14 82.4%	
TNM stage	IVa	43	27 62.8%	0.156	39 90.7%	1.000	28 65.1%	0.157	38 88.4%	0.779
	IVb	17	7 41.2%		16 94.1%		8 47.1%		14 82.4%	

### CD8 and FoxP3 expression are not associated with LDC and survival

Low expression of CD8 or FoxP3 within both stroma and tumor region was scored as 0. High expression of CD8^+^ or FoxP3 in one region and both regions was scored as 1 and 2, respectively. Cancer patients were divided into CD8 or FoxP3 low expression (scored as 0, *n* = 24, 8) groups, median expression (scored as 1, *n* = 22, 9) groups and high expression (scored as 2, *n* = 14, 43) groups.

**Fig. 2:**
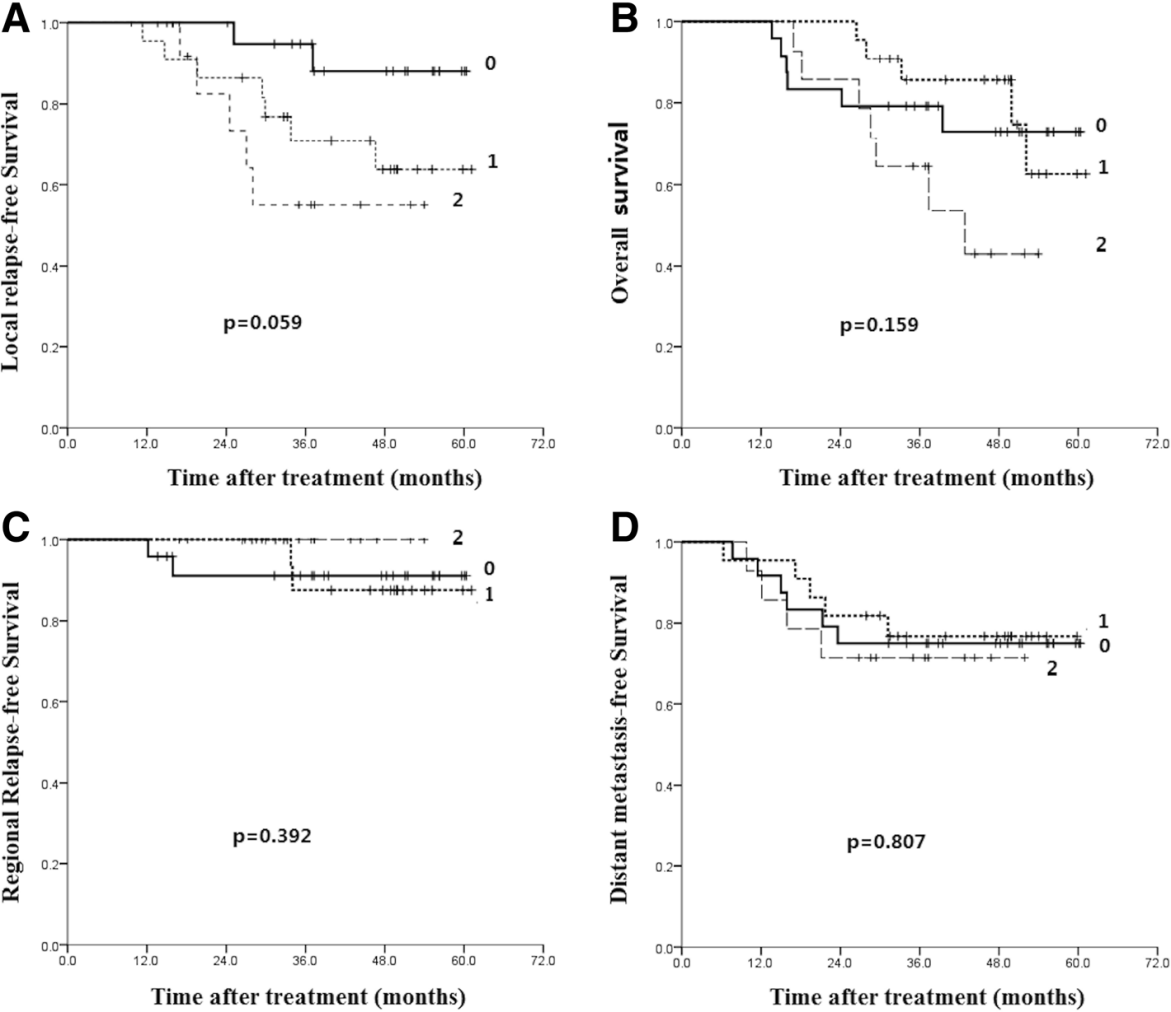
Kaplan-Meier analysis of the correlations between CD8 expression score and patients’ survival: local relapse-free survival (**a**), overall survival (**b**), regional relapse-free survival (**c**) and distant metastasis-free survival (**d**)

The 5-year local relapse-free survival (LRFS) was poorest in CD8 high expression group (*p* = 0.059, Fig. 2a). However, there was no statistical significant difference among three groups, indicating CD8 expression was not associated with LDC. Moreover, there was no significant association between CD8 expression and 5-year OS (75.0, 76.7 and 71.4% for groups scored as 0, 1 and 2, *p* = 0.882) of advanced NPC (Fig. 2b). The similar results were observed in the analysis of 5-year regional relapse-free survival (RRFS) and distant metastasis-free survival (DMFS) (Fig. 2c and d).

**Fig. 3:**
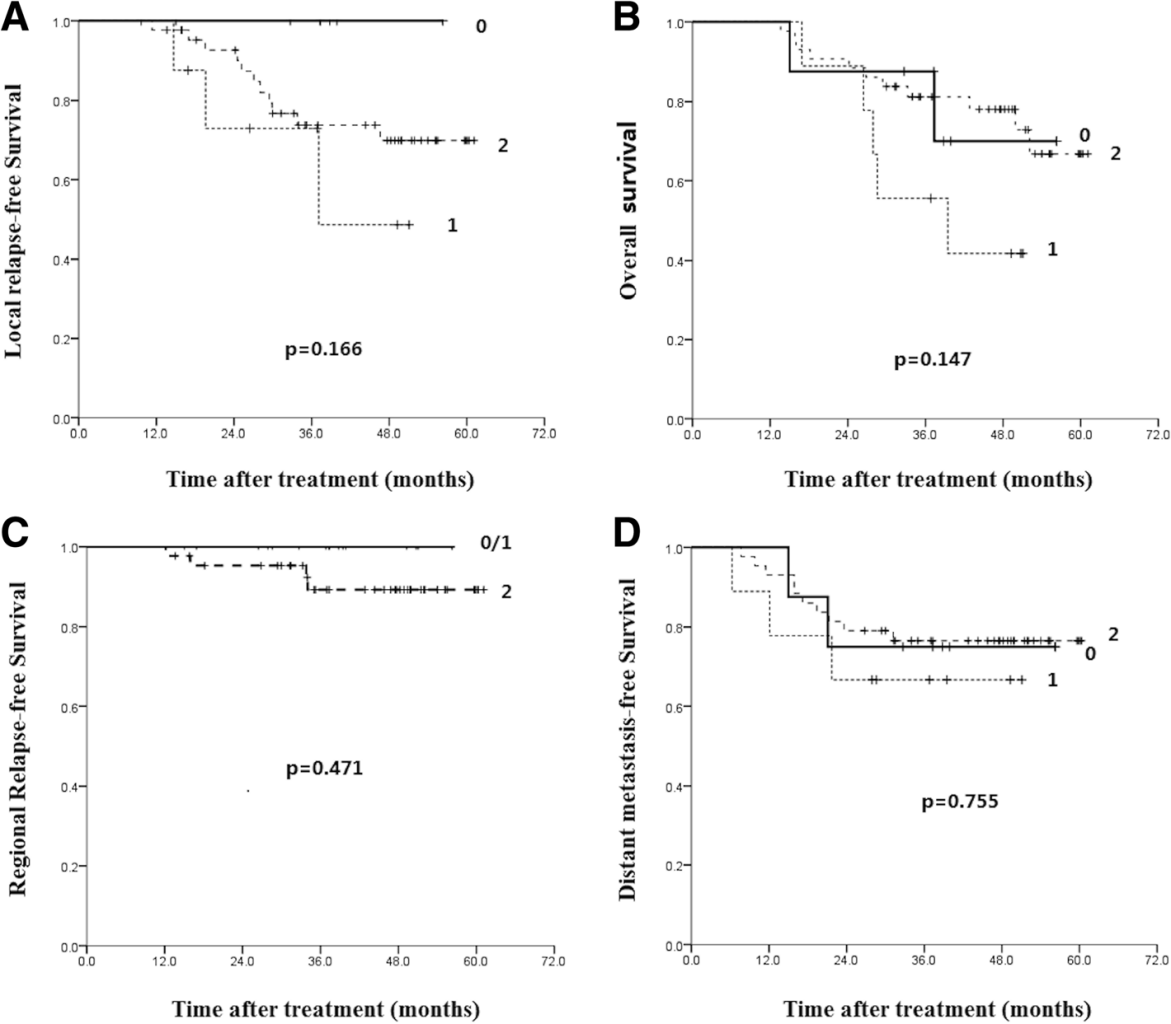
Kaplan-Meier analysis of the correlations between FoxP3 expression score and patients’ survival: local relapse-free survival (**a**), overall survival (**b**), regional relapse-free survival (**c**) and distant metastasis-free survival (**d**)

We also evaluated the correlation between FoxP3 expression and 5-year LDC and survival. Results indicated that low FoxP3 expression showed worst 5-year LRFS (*p* = 0.116, Fig. 3a) in spite of no statistical significant difference. Similar to CD8 results, FoxP3 expression has no significant association with 5-year OS, RRFS and DMFS (Fig. 3b, c and d). Taken together, these results demonstrate that CD8 and FoxP3 expression are not associated with 5-year LDC and survival of stage IVa-b NPC patients.

### PD-1 expression on TILs predicts lower LDC and unfavorable survival

Using the X-Tile statistical package, the optimal cut-off point of PD-1 positive percentage was 5%, and the patients was divided into high expression group (PD-1^+^% > 5%, *n* = 34) and low expression group (PD-1^+^% ≤ 5%, *n* = 26).

**Fig. 4:**
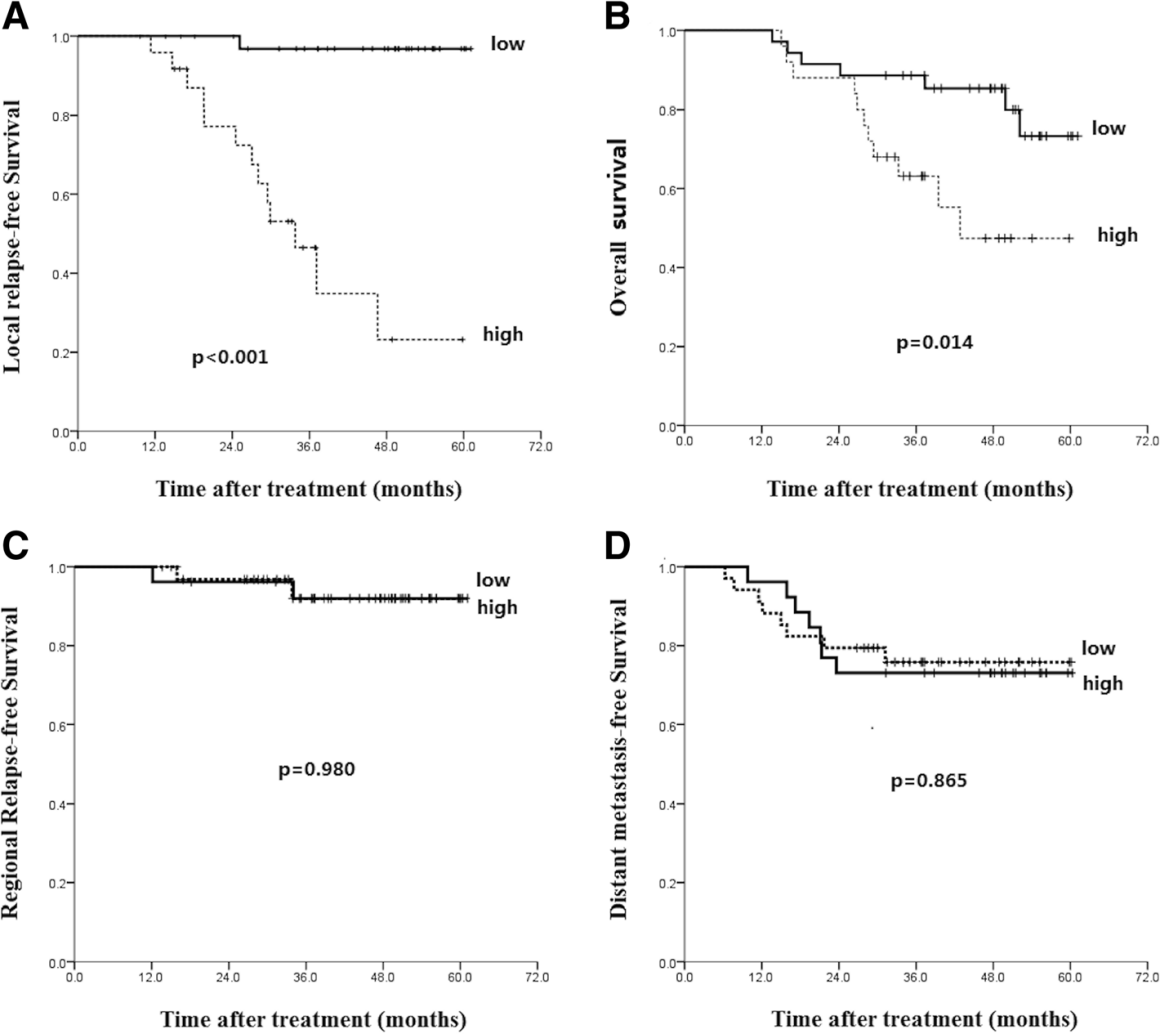
Kaplan-Meier analysis of the correlations between PD-1 expression level and patients’ survival: local relapse-free survival (**a**), overall survival (**b**), regional relapse-free survival (**c**) and distant metastasis-free survival (**d**)

The 5-year LRFS of patients with PD-1 low and high expression was 96.8% and 23.2%, respectively (*p* < 0.001, Fig. 4a), and corresponding 5-year OS was 73.3% vs 47.4% (*p* = 0.014, Fig. 4b). Patients with PD-1 high expression were correlated with lower LDC and OS. However, PD-1 expression has no significant correlation with 5-year RRFS (*p* = 0.980) and DMFS (*p* = 0.865) (Fig. 4c and d).

In multivariate analysis, PD-1 expression was also an adverse prognostic factor for 5-year OS and LRFS (Table 2). The HR of death for high PD-1 expression patients is 3.68 folds higher than those with low PD-1 expression (95% CI: 1.20–11.28, *P* = 0.023). The HR of local relapse for high PD-1 expression patients is 16.89 folds higher than those with low PD-1 expression (95% CI: 1.27–11.84, *P* = 0.007). Other proposed prognostic factors such as age, gender, T stage, N stage, induction chemotherapy and concurrent chemotherapy were analyzed (Table 2).

**Table 2:** Multivariate cox regression analyses estimating the associations of factors with patient survival

Variables	OS		LRFS	
	Exp (B) (95.0% CI)	*P* value	Exp (B) (95.0% CI)	*P* value
PD-1 expression	3.87 (1.27–11.84)	0.018	16.89 (1.27–11.84)	0.007
High vs low				
T stage	1.36 (0.69–2.67)	0.374	4.50 0.66–30.85	0.126
N stage	1.41 (0.81–2.47)	0.224	–	–
Induction chemotherapy	1.53 (0.51–4.56)	0.449	5.89 (0.74–46.68)	0.094
No vs yes				
Concurrent chemotherapy No vs yes	1.416 (1.17–12.09)	0.750	2.899 (0.36–23.28)	0.317
Gender	0.69 (0.19–2.57)	0.579	0.85 (0.18–4.03)	0.835
Female vs male				
Age	0.68 (0.25–1.88)	0.460	0.70 (0.40–1.25)	0.225
< 47 vs ≥47				
Pathology	0.65 (0.08–5.39)	0.692	0.379 (0.08–1.86)	0.232
Non-keratinizing vs keratinizing				

**Fig. 5:**
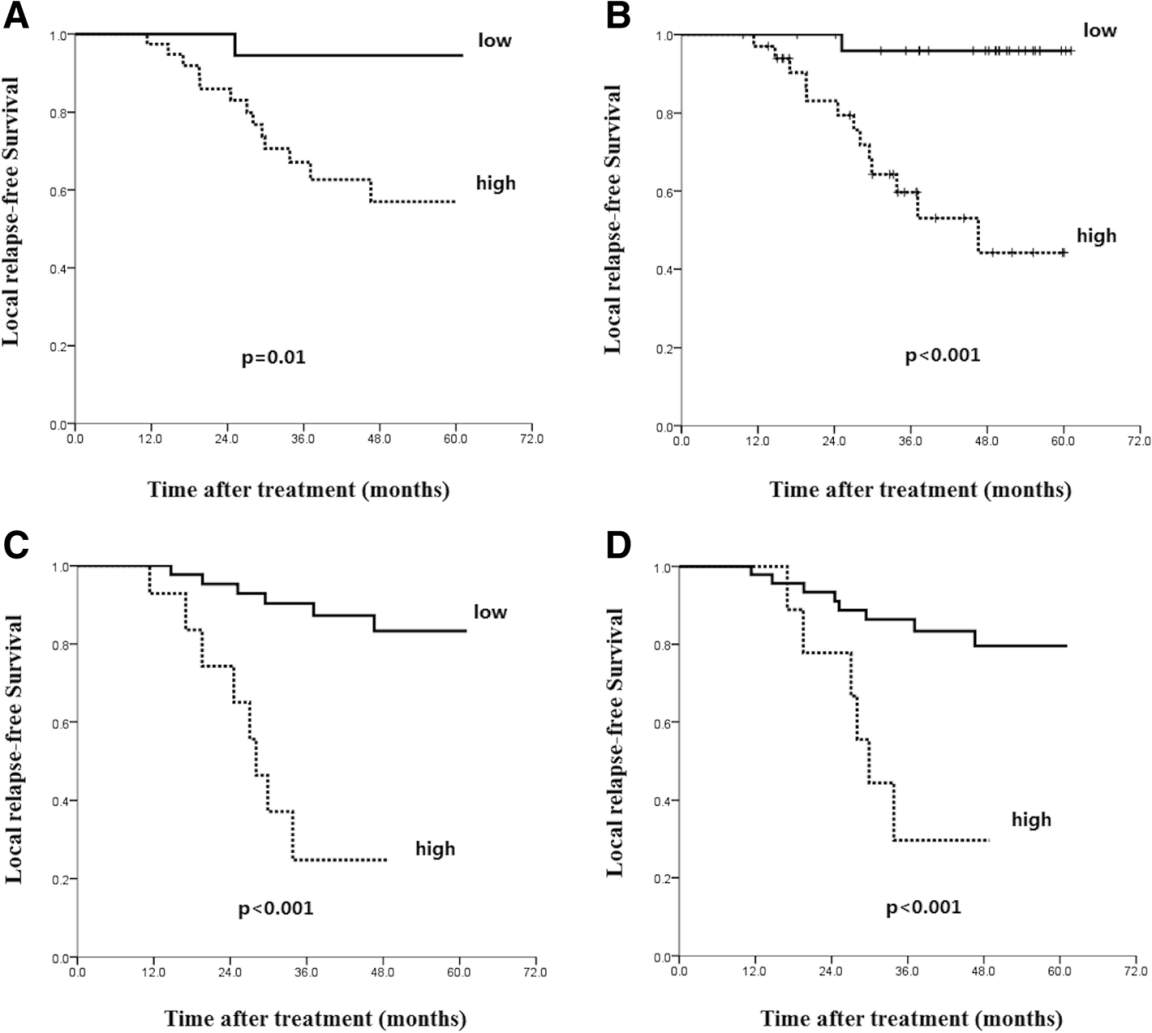
Kaplan-Meier analysis of the correlations between PD-1 expression level and patients’ local relapse-free survival with cut-off points at 1% (**a**), 5% (**b**), 10% (**c**) and 15% (**d**)

Cut-off points of 1%, 10% and 15% in addition to 5% were also used for the analysis of PD-1 expression on local disease control. As shown in Fig. 5, the same trend of PD-1 expression on local disease control was evident.

**Fig. 6:**
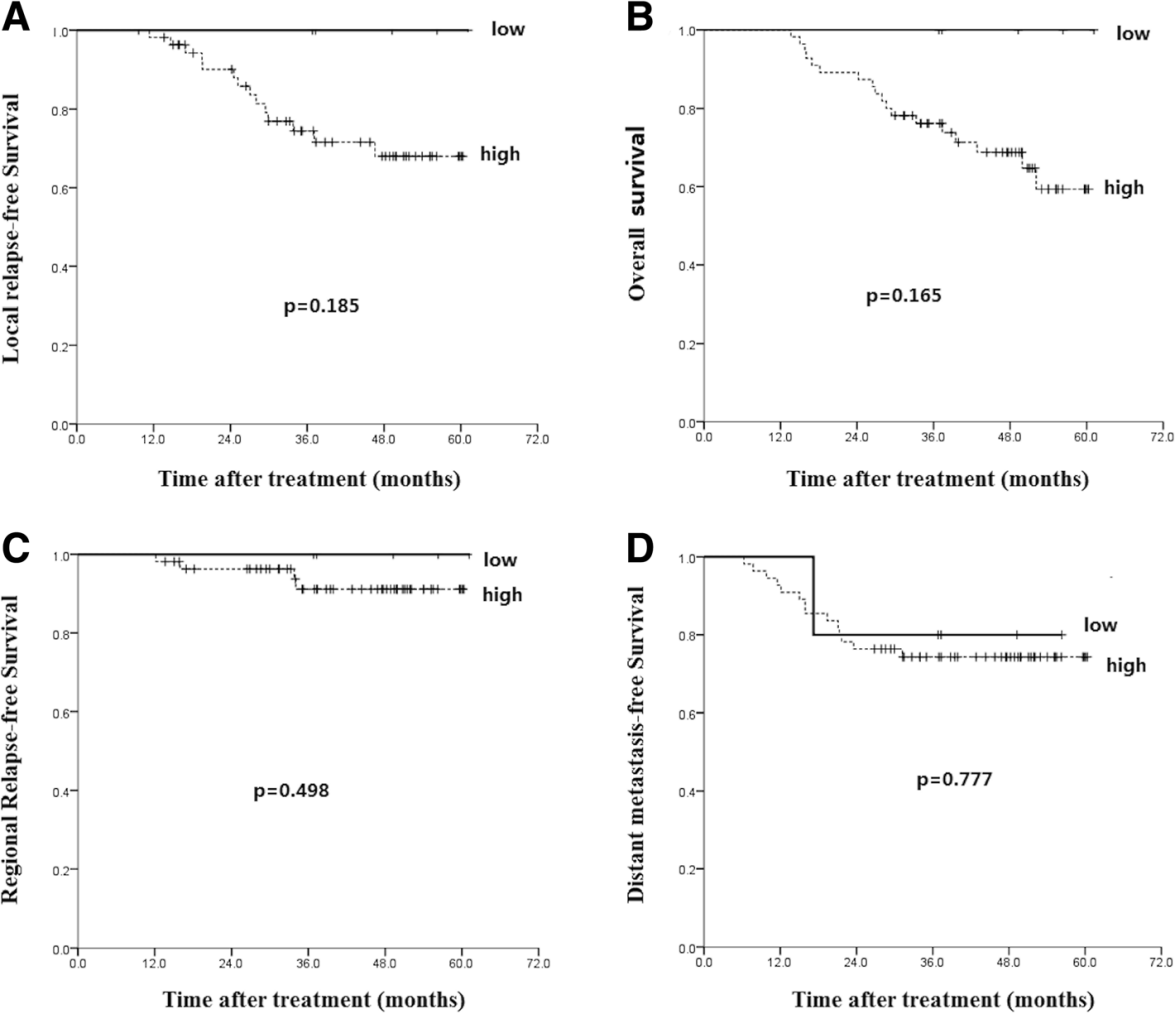
Kaplan-Meier analysis of the correlations between PD-L1 expression level and patients’ survival: local relapse-free survival (**a**), overall survival (**b**), regional relapse-free survival (**c**) and distant metastasis-free survival (**d**)

Then we analyzed the effect of PD-L1 expression on LDC and survival. Despite all the 5 patients with low PD-L1 level are alive, survival analysis showed that no difference of 5-year LRFS (*p* = 0.185), OS (*p* = 0.165), RRFS (*p* = 0.498) and DMFS (*p* = 0.777) was found between low and high expression level of PD-L1 (Fig. 6). These findings indicate that PD-1 expression but not PD-L1 was associated with lower 5-year LDC and unfavorable 5-year OS.

### Influence of PD-1 distribution on the prognosis of NPC

Next, we determined the correlation of PD-1 distribution with prognosis. We divided the patients into Group A with both stromal and intratumoral PD-1 low expression (*n* = 26), Group B with both stromal and intratumoral PD-1 high expression (*n* = 9), and Group C with stromal PD-1 high expression only (*n* = 25). There is no patient with only intratumoral PD-1 high expression.

**Fig. 7:**
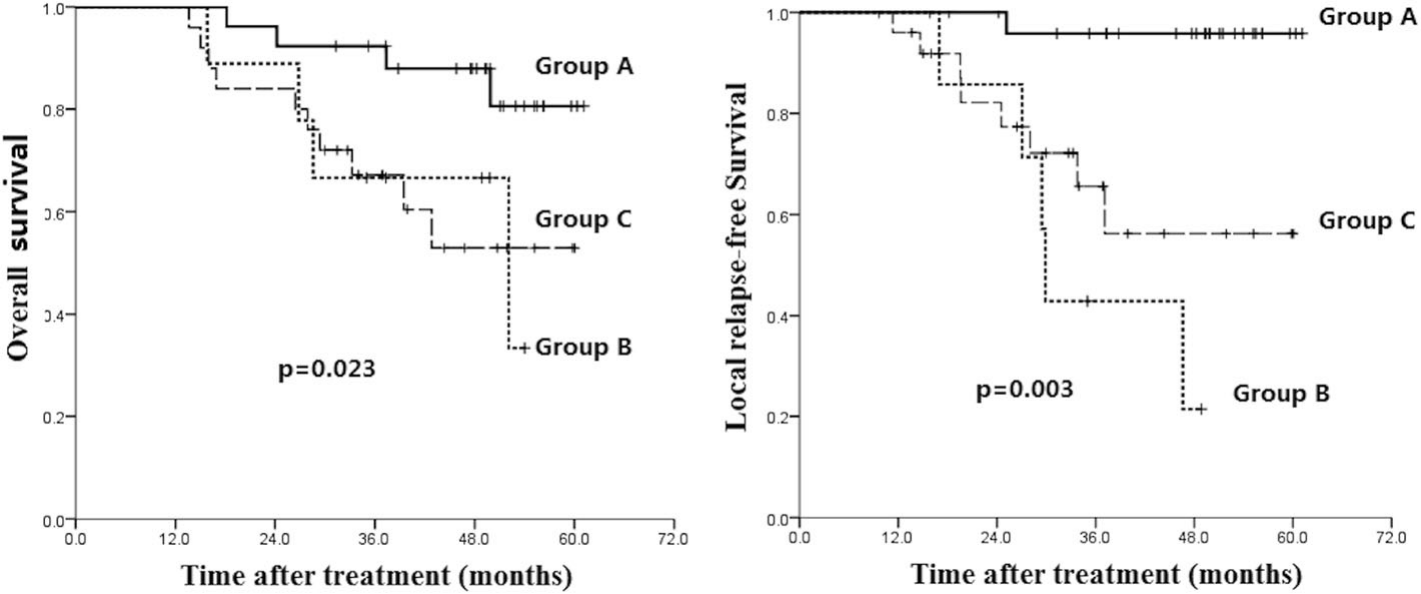
Kaplan-Meier analysis of the correlation between different PD-1 expression pattern and post-treatment survival. Group A: stromal PD-1^low^/intratumoral PD-1^low^; Group B: stromal PD-1^high^/intratumoral PD-1^high^; Group C: stromal PD-1^high^/intratumoral PD-1^low^

As shown in Fig. 7, both group B and C had a significant inferior LRFS (*p* = 0.003) and OS (*p* = 0.023) compared with group A. Patients in group B showed the worst LDC and survival (5-year LRFS 21.4% and 5-year OS 34.6%) compared with group C (5-year LRFS 56.2% and 5-year OS 56.4%) and Group A (5-year LRFS 95.8% and 5-year OS 82.2%). These data suggest that PD-1 distribution in both stroma and tumor region predicts the poorest prognosis.

### PD-1/PD-L1 pathway activation predicts lower LDC and unfavorable survival

**Fig. 8:**
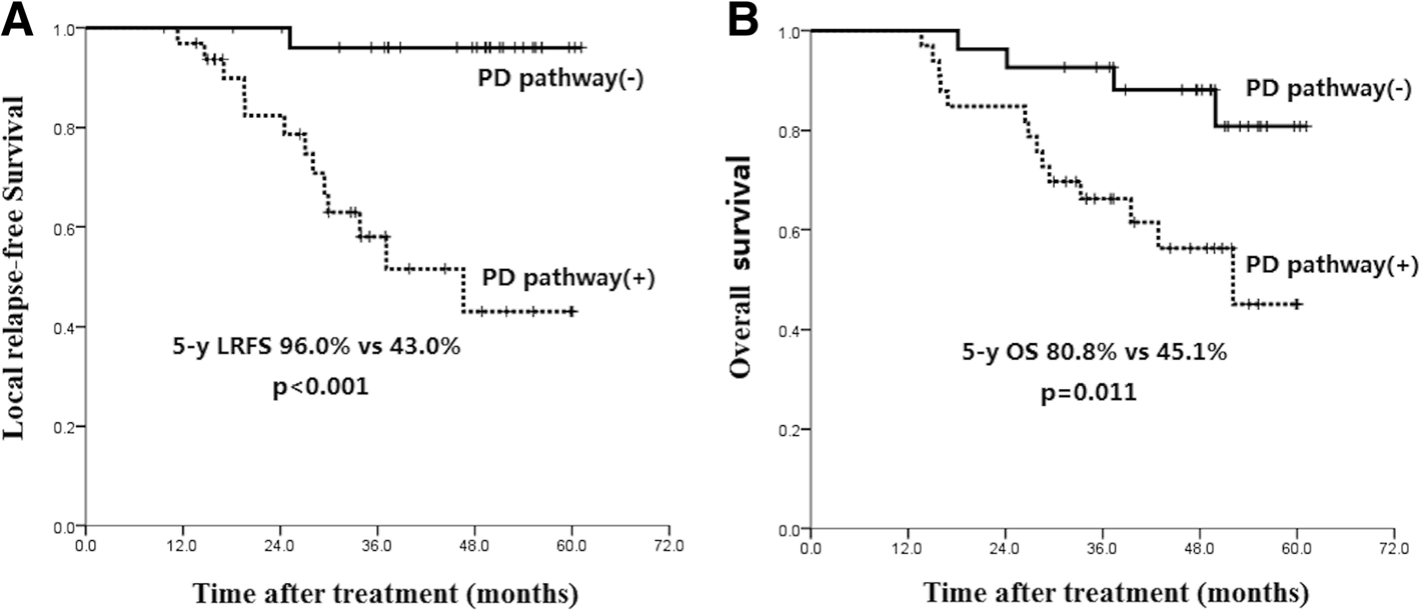
Kaplan-Meier analysis of the correlations between PD-1/PD-L1 pathway activation and patients’ survival: local relapse-free survival (**a**), overall survival (**b**)

The patients were first classified as the following groups: both PD-1 and PD-L1 high expression (*n* = 27), PD-1 high expression and PD-L1 low expression (*n* = 1), PD-1 low expression and PD-L1 high expression (*n* = 28), and both PD-1 and PD-L1 low expression (*n* = 4). It’s found that patients in the group with both PD-1 and PD-L1 high expression had a significant lower local relapse-free survival (5-year LRFS 29.9%), while the other groups had similar 5-year LRFS (100%, 95.8% and 100%, respectively). Then the patients were divided to two groups: PD-1/PD-L1 pathway activated, i.e. both PD-1 and PD-L1 high expression (n = 27), and PD-1/PD-L1 pathway inactivated (*n* = 33). Patients with activated PD-1/PD-L1 pathway had a significant poor local disease control (5-year LRFS 96.0% vs 43.0%, *p* < 0.001, Fig. 8a) and overall survival (5-year OS 80.8% vs 45.1%, p < 0.001, Fig. 8b) than those with unactivated pathway.

## Discussion

In this study, we demonstrate that PD-1 high expression, especially combined with PD-L1 high expression, is associated with lower 5-year LDC and unfavorable 5-year OS of stage IV M0 NPC patients, predicting higher local recurrence rate and poorer clinical outcome. While, there was no statistical significant correlation between CD8, FoxP3, and PD-L1 expression and the patient prognosis.

A high density of CD8^+^ T cells has been reported to be associated with improved outcome of various tumors [28, 29]. While, intratumoral CD8^+^ T cells with high PD-1 expression predicted poor prognosis of NPC [22]. Our data showed that CD8^+^ density is not an independent prognostic factor for LRFS and OS. However, CD8^+^ T cell counts tended to be correlated with lower LDC and poorer outcome. We also found that patients with high density of CD8^+^ T cells showed elevated expression level of PD-1. Thus we speculated that these tumor infiltrating CD8^+^ T cells may express high level PD-1 and lead to impairment of function.

In the present study, high PD-1 expression on TILs was found in 56.7% of the stage IV M0 patients. Zhou et al. [30] reported a corresponding rate of 44%. While in Larbcharoensub’s work [31], they observed PD-1 expression in tumor cells instead of TILs, 11% of tumors expressed PD-1. High expression of PD-1 was found to be correlated with lower LDC and poor OS. Moreover, patients with PD-1 high expression at both stroma and intratumor region were associated with even poorer LDC and OS when compared to those had PD-1 high expression at eithor stroma or intratumor region, while patients with both regional PD-1 low expression had the best LDC and OS. To our knowledge, the question of PD-1 expression with nasopharyngeal carcinoma local control and overall survival has rarely been addressed. In a study by Hsu et al. [22], 46 cases of nasopharyngeal carcinoma were analyzed, and higher expression of PD-1 was found to be correlated with a poorer prognosis of locoregional recurrence-free survival, disease-free survival, and overall survival. On the contrary, in a recent study by Zhou et al. [30], using a cut-off point of 5%, PD-1 expression was reported to be of no significant association with OS or PFS. However, majority of the patients (65/99, 66%) were of staging I-III, and a high 3-year OS of 94.9% was reported, which may blur the effects of PD-1 expression. In another study by Zhang et al. [21], PD-1 expression was not a prognostic factor. Similar to Zhou’s report, only about 30% of the NPC patients were of stage IV disease. Taken together, high PD-1 expression seems to be an adverse factor for nasopharygeal carcinoma local control and survival, especially for patients with late-stage cancers.

We found that PD-L1 was highly expressed in 91.7% of stage IVa-b NPC patients, which is in line with the previous results (95% and 97%) in NPC [21, 30], and similar with reports in the other malignancies [32–35]. However, much lower expression rate (25%) in the NPC patients was also reported [36]. Though there is no statistical association between PD-L1 expression and the prognosis of NPC patients, all 5 patients with low PD-L1 expression were alive without local recurrent evidence after 37–61 months of follow-up. In the report by Zhou et al., a high expression of PD-L1 was correlated with shorter OS and showed trend of a reduced PFS rate [30]. The study conducted by Zhang [21] also showed that patients with high expression of PD-L1 were correlated with more poor PFS. Inversely, Lee et al. revealed that PD-L1 high expression was associated with better LRRFS and PFS [36]. Further study with larger samples was warranted.

PD-1/PD-L1 is considered to be a crucial immune checkpoint mediating cancer immune escape due to T cell dysfunction [37]. The activation of this pathway needs both PD-1 expression in T cells and its ligand expression in the tumor cells. We found that activated PD-1/PD-L1 pathway was an independent negative indicator for local disease control (HR = 31.73, *P* = 0.001) and overall survival (HR = 3.37, *P* = 0.017). For the first time, we identify the relationship between the PD-1/PD-L1 pathway activation and therapeutic outcome in nasopharyngeal carcinoma.

High infiltration of FoxP3^+^ Tregs has been reported to be associated with unfavorable outcome of multiple malignancies including breast cancer [38], ovarian carcinoma [39], lung cancer [40], hepatocellular carcinoma [41], and gastric cancer [42]. However, other studies reported that increased frequency of FoxP3^+^ Tregs was associated with improved prognosis in colorectal cancer [43] and head and neck squamous cell carcinoma [44]. Our results showed that high expression of FoxP3^+^ has no significant correlation with NPC prognosis, indicating that the prognostic value of FoxP3^+^ Tregs might depends on specific cancer type.

Although recommendations for the evaluation of TILs in breast cancer had been proposed by an International TILs Working Group 2014 [45], there was no standard scoring system to describe TILs in head and neck cancers. In the few reports concerning nasopharyngeal carcinoma, the methods used for TILs evaluation were different [20–22, 30, 31]. In the reports by Hsu [22] and Larbcharoensub [31], PD-1 expression was scored as the percentage of the immune cells with positive staining. In the other reports [20, 21, 30], the percentage of PD-1 stained TILs and staining intensity was evaluated. However, the details of TILs assessment were not described. In our work, mononuclear cells around tumor nests and not directly contacting carcinoma cells was categorized as stromal TILs, and those within tumor nests directly interacting with carcinoma cells as intratumoral TILs. This was in accordance with the principles suggested by the International TILs Working Group 2014. Consensus for TILs assessment in nasopharyngeal carcinoma is urgently needed. A standardized evaluation system will help to overcome barriers to clinical implementation.

Our study is limited by a relatively small sample size, only 60 stage IV M0 patients were included. The disease-free survival was set as the outcome to estimate the optimal cut-off point of the biomarkers. Taking PD-1 for example, 5% was given by the X-tile as the optimal cut-off value. In addition to 5%, cut-off points at 1%, 10% and 15% were also tried to explore the impact of PD-1 expression on the disease control. Although the same tendency was evident at all these mentioned cut-off points, suitable cut-off value should be validated in a larger cohort of patients before being used as a predefined cut-off in the following clinical trials. Moreover, standard experiment procedures and scoring criteria for the biomarkers are urgently needed.

## Conclusions

The present study revealed that PD-1 high expression, especially with PD-L1 co- expression, is associated with high local recurrence and unfavorable clinical outcome of stage IV M0 NPC patients. Therefore, PD-1/PD-L1 pathway might be a biomarker to identify patients prone to therapeutic failure and poor prognosis. Clinical trials with combination of PD-1/PD-L1 inhibitor and chemoradiotherapy are warranted for those patients.

## Abbreviations

DMFS: Distant metastasis-free survival
HNSCC: Head-and-neck squamous carcinoma
IMRT: Intensity-modulated radiotherapy
LDC: Local disease control
LRFS: Local relapse-free survival
NPC: Nasopharyngeal carcinoma
OS: Overall survival
PD-1: Programmed death-1
PD-L1: Programmed death-ligand 1
PFS: Progression-free survival
RRFS: Regional relapse-free survival
TAMs: Tumor associated macrophages
TEM: Tumor microenvironment
TILs: Tumor-infiltrating lymphocytes
Tregs: Regulatory T lymphocytes
